# Group antenatal care for improving retention of adolescent and young pregnant women living with HIV in Kenya

**DOI:** 10.1186/s12884-022-04527-z

**Published:** 2022-03-15

**Authors:** Chloe A. Teasdale, Judith Odondi, Catherine Kidiga, Michelle Choy, Ruby Fayorsey, Bernadette Ngeno, Boniface Ochanda, Agnes Langat, Catherine Ngugi, Tegan Callahan, Surbhi Modi, Mark Hawken, Doris Odera, Elaine J. Abrams

**Affiliations:** 1 Department of Epidemiology & Biostatistics, City University of New York (CUNY) Graduate School of Public Health & Health Policy, 55 W125th Street, Room 543, New York, NY 10025 USA; 2 ICAP-Columbia University, Mailman School of Public Health, New York, NY USA; 3 Department of Epidemiology, Mailman School of Public Health, Columbia University, New York, NY USA; 4 Division of Global HIV and TB, US Centers for Disease Control and Prevention, Atlanta, GA USA; 5 US Centers for Disease Control and Prevention, Nairobi, Kenya; 6 National AIDS and STI Control Programme (NASCOP), Nairobi, Kenya; 7 Department of Pediatrics, Vagelos College of Physicians & Surgeons, Columbia University, New York, NY USA

**Keywords:** Prevention of mother-to-child transmission of HIV, Group antenatal care, Retention, Antiretroviral therapy, Adolescent pregnancy

## Abstract

**Background:**

Pregnant and breastfeeding adolescents and young women living with HIV (AYWLH) have lower retention in prevention of mother-to-child transmission (PMTCT) services compared to older women.

**Methods:**

We evaluated a differentiated service model for pregnant and postnatal AYWLH at seven health facilities in western Kenya aimed at improving retention in antiretroviral treatment (ART) services. All pregnant AYWLH < 25 years presenting for antenatal care (ANC) were invited to participate in group ANC visits including self-care and peer-led support sessions conducted by health facility nurses per national guidelines. ART register data were used to assess loss to follow-up (LTFU) among newly-enrolled pregnant adolescent (< 20 years) and young women (20–24 years) living with HIV starting ART in the pre-period (January-December 2016) and post-period (during implementation; December 2017-January 2019). Poisson regression models compared LTFU incidence rate ratios (IRR) in the first six months after PMTCT enrollment and risk ratios compared uptake of six week testing for HIV-exposed infants (HEI) between the pre- and post-periods.

**Results:**

In the pre-period, 223 (63.2%) of 353 pregnant AYWLH newly enrolled in ANC had ART data, while 320 (71.1%) of 450 in the post-period had ART data (*p* = 0.02). A higher proportion of women in the post-period (62.8%) had known HIV-positive status at first ANC visit compared to 49.3% in the pre-period (*p* < 0.001). Among pregnant AYWLH < 20 years, the incidence rate of LTFU in the first six months after enrollment in ANC services declined from 2.36 per 100 person months (95%CI 1.06–5.25) in the pre-period to 1.41 per 100 person months (95%CI 0.53–3.77) in the post-period. In both univariable and multivariable analysis, AYWLH < 20 years in the post-period were almost 40% less likely to be LTFU compared to the pre-period, although this finding did not meet the threshold for statistical significance (adjusted incidence rate ratio 0.62, 95%CI 0.38–1.01, *p* = 0.057). Testing for HEI was 10% higher overall in the post-period (adjusted risk ratio 1.10, 95%CI 1.01–1.21, *p* = 0.04).

**Conclusions:**

Interventions are urgently needed to improve outcomes among pregnant and postnatal AYWLH. We observed a trend towards increased retention among pregnant adolescents during our intervention and a statistically significant increase in uptake of six week HEI testing.

## Background

Adolescents (15–19 years) and youth (20–24 years) living with HIV are less likely to be on antiretroviral treatment (ART) and to be retained in care compared to both children and adults living with HIV [1–5]. Pregnant and postpartum adolescents (< 20 years) and young (20–24 years) women living with HIV (AYWLH) are at higher risk for being lost from antenatal care (ANC) and ART services, and are less likely to receive the full package of prevention of mother-to-child HIV transmission (PMTCT) interventions compared to older women living with HIV (WLHIV) [6–10]. A study in Kenya found that, compared to pregnant adult women living with HIV (≥ 20 years), adolescents attended fewer antenatal visits, were less likely to be on ART, and fewer of their infants received HIV-prophylaxis [6].

In order to maintain the health of AYWLH and to prevent MTCT, it is critical that all pregnant AYWLH receive the full package of PMTCT services, including HIV testing and prophylaxis for HIV-exposed infants (HEI) and continued lifelong ART for mothers. While approaches to improve PMTCT retention have been identified [11, 12], there has been little research into interventions specifically targeted to the needs of pregnant and postpartum AYWLH [9]. Barriers to ART retention in the general adolescent population include fear of disclosure, social isolation and challenging relationships with healthcare workers [13, 14]. For pregnant AYWLH, these may be compounded by receipt of ANC services in clinics designed for adults.

There is an urgent need to identify differentiated service delivery (DSD) approaches that improve outcomes in pregnant and postpartum AYWLH. The group ANC model, which aims to build peer support and reduce feelings of isolation, is an intervention that has been shown to improve ANC retention and pregnancy outcomes among adolescents [15, 16]. There are no known previous studies of group antenatal care in the context of PMTCT services for pregnant and postpartum AYWLH. We implemented and evaluated a DSD model of enhanced PMTCT services, including group ante- and post-natal care, aimed at improving ART retention and uptake of testing for HEI among pregnant and postpartum AYWLH in western Kenya.

## Methods

### Intervention

Project HOPE was developed by the Kenyan National AIDS and STI Control Program (NASCOP), the US Centers for Disease Control and Prevention (CDC), and ICAP at Columbia University to strengthen and enhance existing services for ANC, PMTCT and HEI care and testing among AYWLH. It was implemented from December 2017 through January 2019 at seven health facilities in western Kenya (purposively selected based on high patient volume). All aspects of standard of care (SOC) ANC, PMTCT and postnatal clinical services as per Kenya national guidelines were included in the care model. Kenya 2016 national guidelines call for initiation of lifelong ART at HIV diagnosis for all people living with HIV, including pregnant and breastfeeding women, integrated ANC and ART services for mother and infants within maternal child health (MCH) clinics for 24 months, and polymerase chain reaction (PCR) HIV testing at 6–8 weeks of life for HEI [17]. In 2014, Kenya introduced the Adolescent’s Package of Care which includes information and resources for health care workers who provide care to adolescents on best practices for HIV prevention and care and treatment services [18].

**Fig. 1:**
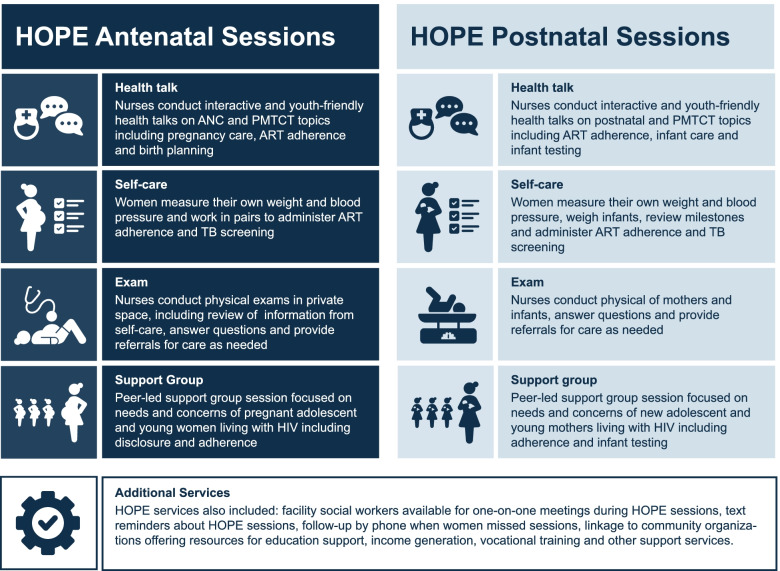
Project HOPE group care model for adolescent and young pregnant women living with HIV developed by ICAP at Columbia University

As part of the HOPE DSD model, all pregnant AYWLH attending ANC at participating facilities were asked to come to monthly group ANC visits, or “HOPE sessions” through six months postpartum, instead of individual care visits (women declining HOPE sessions could attend individual ANC visits). HOPE sessions were held according to monthly schedules at each facility and included the services in Fig. 1. Women graduated out of HOPE services and returned to SOC when their infants reached six months.

At each health facility, up to three nurses and a mentor mother (recently pregnant AYWLH) were trained by the study team and oversaw HOPE sessions with bimonthly visits from clinical mentors. At the start of the project, all pregnant AYWLH who were already attending services at the health facility were invited to attend their next ANC visit during a HOPE session; all postpartum AYWLH who had delivered in the previous three months were invited to join separate postnatal HOPE sessions (there were no exclusion criteria). The model was designed for a maximum of 13 women per session; at larger facilities, multiple HOPE ANC and postnatal groups were held per month. Groups were designed to include women who were of (roughly) the same gestational age and women remained in the same groups throughout their pregnancies. Consent was not required as the facility offered the service as a new model of care for all pregnant AYWLH.

### Data sources

Routinely-collected facility data were reviewed to compare outcomes among women attending ANC at the seven project health facilities during the HOPE project (December 2017 through January 2019; “post-period” with data collection through June 2019) to the outcomes of a cohort of women who attended care at the same facilities prior to the project (January through December 2016; “pre- period”). The evaluation included women newly enrolled in ANC and starting ART during the pre- and post-periods with documented ART data at the facility. Women already enrolled in ANC services when the project started were excluded from the evaluation, as were women not found in the ART register. Trained data collectors abstracted information from the national paper-based ANC, ART and HEI registers at the end of implementation. Data from ANC registers included gestational age, parity, marital status and known or new HIV diagnosis at first ANC visit. ART register data provided initiation date, regimen, months of ART pick-up, and documentation of: lost to follow-up (LTFU), transfer to another facility, death, or stopped ART. Descriptive characteristics of women in the pre- and post-period cohorts disaggregated by age group (< 20 vs. 20–24 years) were compared using Cochran-Mantel–Haenszel (adjusted for facility) and Wilcoxon tests.

### Evaluation and statistical analyses

The primary outcome was the incidence rate of LTFU in the first six months after enrollment in ANC services among women who initiated ART. Person-time was estimated using first ANC visit date and last ART pick-up date (last day of last month woman received ART). Women with a date of transfer to another facility or death were censored at last ART pick-up and those with less than six months of ART data missing transfer or death date were considered LTFU. Poisson regression was used to estimate incidence rates for LTFU in the first six months after enrollment in PMTCT services in the pre- and post-period cohorts. To assess whether the HOPE intervention reduced LTFU, multivariable Poisson models were used to compare incidence rate ratios (IRR) of LTFU between the pre- and post-cohorts for women within age groups. Models were adjusted for a priori determined predictors of retention in PMTCT: gestational age (weeks), parity, known HIV status at enrollment in ANC and already on ART at first ANC visit. For the main analysis comparing the pre- and post-period cohorts, we included all newly enrolled women in the post-period cohort, including those who never attended a HOPE session consistent with an intent-to-treat analysis. We also conducted a pre- and post-period comparison using the subset of women from the post-period who attended at least one HOPE session.

The proportion of women whose infants received early infant HIV diagnostic (EID) testing at 6–8 weeks and test results were compared between the pre- and post-periods using data abstracted from the paper-based HEI registers. Women in the post-period who had not delivered or whose infants were not estimated to have reached 6–8 weeks of age at data collection were excluded (date of delivery was not available; infant age based on mother’s gestational age at first ANC visit; not accounting for pregnancy loss or multiple births). Modified Poisson relative risk (RR) regression models with robust standard errors were used to compare HEI testing at 6–8 weeks between the pre- and post-period adjusted for the same covariates noted above. All models were adjusted for intra-site clustering across the seven facilities. Analyses were conducted in SAS 9.4 (SAS Institute Inc., Cary,NC,USA).

This study was reviewed and approved by the CDC Institutional Review Board (IRB) (protocol #7011.0) and approved by the Columbia University Irving Medical Center (CUIMC) IRB and the Kenya Medical Research Institute (KEMRI). A waiver of consent was granted by the CUIMC IRB and the KEMRI ethics boards for use of routinely collected retrospective data for the evaluation. All methods were performed in accordance with the relevant guidelines and regulations (Declaration of Helsinki).

## Results

### Participant characteristics pre/post

**Fig. 2:**
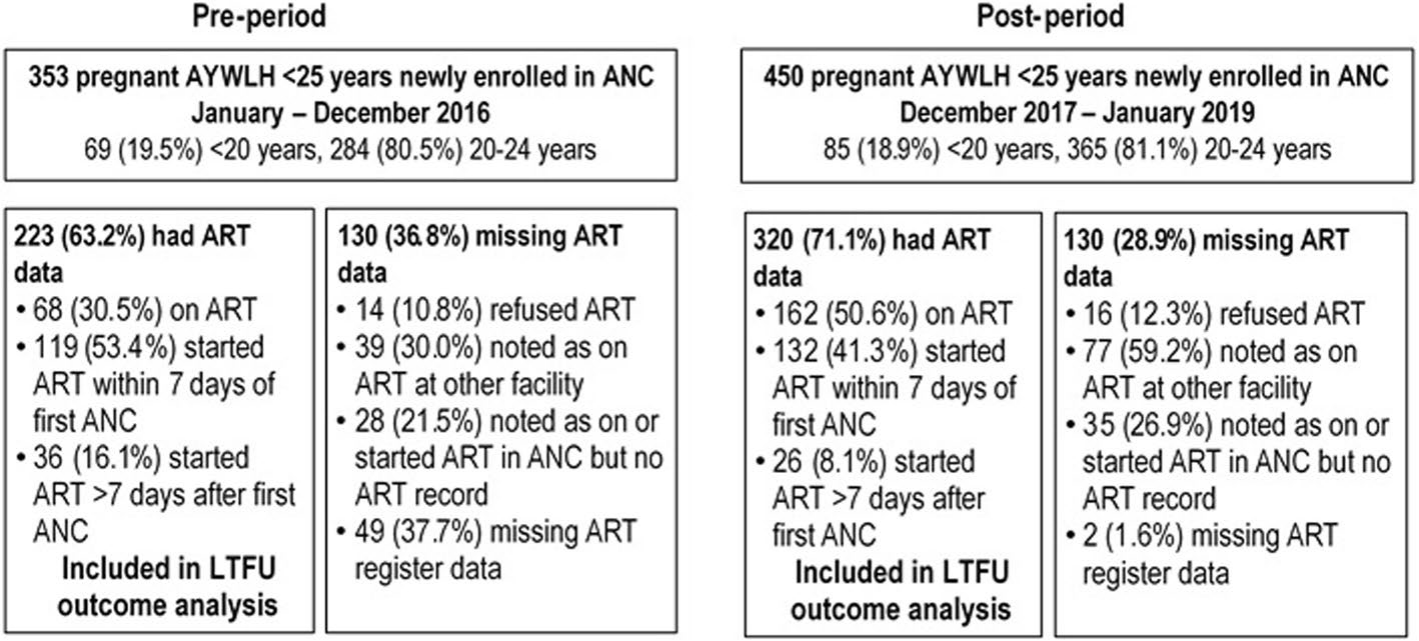
Antiretroviral treatment (ART) status information in antenatal care (ANC) among newly enrolled pregnant adolescent and young women living with HIV (AYWLH) < 25 years of age in the pre- and post-periods. ANC: antenatal care; ART: antiretroviral therapy; LTFU: loss to follow-up

In the pre-period, 223 (63.2%) of 353 pregnant AYWLH newly enrolled in ANC at the seven project health facilities had ART data. Of the 223 AYWLH with ART data, 68 (30.5%) were on ART prior to the first ANC visit, 119 (53.4%) started ART within 7 days of the first ANC visit, and 36 (16.1%) started ART > 7 days after the first ANC visit (Fig. 2). Among the 130 (36.8%) women missing ART initiation dates, 14 (10.8%) refused ART, 39 (30.0%) were noted as being on ART at another facility, 28 (21.5%) were indicated as being on or starting ART but were not found in the ART register and 49 (37.7%) had no information about ART status in either the ANC or ART registers. In the post-period 450 pregnant AYWLH were newly enrolled in ANC, among whom 320 (71.1%) had ART data; 162 (50.6%) were on ART at the first ANC visit, 132 (41.3%) started ART within 7 days, and 26 (8.1%) started > 7 days after first ANC (pre vs. post-period proportion of women with ART data *p* = 0.02). Of the 130 women missing ART initiation dates, 16 (12.3%) refused ART, 77 (59.2%) were indicated to be on ART at another facility, 35 (26.9%) were noted as on ART or started ART but were missing an ART record and 2 (1.6%) women had no information (Fig. 2).

Of the 223 women with ART data included in the analysis from the pre-period, 47 (21.1%) were < 20 years of age and 176 (78.9%) were 20–24 years of age, while in the post-period cohort of 320 women with ART data, 53 (16.6%) were < 20 years and 267 (83.4%) were 20–24 years (Table 1). Median gestational age at first ANC visit was 22 weeks (interquartile range (IQR) 16–26) in the pre-period and 20 weeks (IQR 14–26 in the post-period (*p*-value = 0.11). Women in the post-period were significantly more likely to have a known HIV diagnosis (49.3% pre, 62.8% post%, *p* < 0.001) and to be on ART at first ANC (30.5% pre, 50.6% post, *p* < 0.0001). Among the 320 women in the post-period cohort, 255 (79.7%) attended at least one HOPE session, median number of visits was 7 (IQR 5–9) (Table 1).

**Table 1:** Characteristics of pregnant adolescent and young women living with HIV (AYWLH) < 25 years of age newly enrolled in antenatal (ANC) services with antiretroviral therapy (ART) start date at seven HOPE project facilities in Kenya in the pre- and post-periods (*N* = 543)

	**Pre-period** AYWLH newly enrolled ANC Jan-Dec 2016						**Post-period (All)** AYWLH newly enrolled ANC Dec 2017-Jan 2019						**Pre- vs. post-period**	**Post-period (HOPE attendees)** AYWLH newly enrolled ANC Dec 2017-Jan 2019						**Pre- vs. post-period**
	** < 20 years**		**20–24 years**		**All**		** < 20 years**		**20–24 years**		**All**			** < 20 years**		**20–24 years**		**All**		
	N	%	N	%	N	%	N	%	N	%	N	%	***p*-value**	N	%	N	%	N	%	***p*-value**
	47	21.1	176	78.9	223	100.0	53	16.6	267	83.4	320	100.0		46	18.0	209	82.0	255	100.0	
**Gestational age weeks, median (IQR)**	22 (16–29)		22 (16–26)		22 (16–26)		22 (14–28)		20 (14–26)		20 (14–26)		0.11	24 (14–28)		20 (13–26)		20 (13–26)		0.16
**Trimester**														12	26.1	57	27.3	69	27.1	
1st	9	19.2	31	17.7	40	18.0	15	28.3	69	25.8	84	26.3	0.06	20	43.5	108	51.7	128	50.2	0.05
2nd	22	46.8	109	62.3	131	59.0	24	45.3	139	52.1	163	50.9		14	30.4	44	21.1	58	22.8	
3rd	16	34.0	35	20.0	51	23.0	14	26.4	59	22.1	73	22.8								
Parity*																				
0	30	63.8	34	19.3	64	28.7	41	77.4	84	31.6	125	39.2	0.06	35	76.1	73	35.1	108	42.5	0.03
1–3	17	36.2	137	77.8	154	69.1	12	22.6	180	67.7	192	60.2		11	23.9	133	63.9	144	56.7	
> 3	0	0.0	5	2.8	5	2.2	0	0.0	2	0.8	2	0.6		0	0.0	2	1.0	2	0.8	
Married (reported)*	30	63.8	152	86.4	182	81.6	32	60.4	214	80.2	246	76.9	0.20	28	60.9	163	78.0	191	74.9	0.10
HIV status at entry to ANC*																				
Newly diagnosed	32	68.1	81	46.0	113	50.7	26	49.1	93	34.8	119	37.2	< 0.001	22	47.8	77	36.8	99	38.8	< 0.01
Known HIV-positive	15	31.9	95	54.0	110	49.3	27	50.9	174	65.2	201	62.8		24	52.2	132	63.2	156	61.2	
**ART status from ANC register**																				
On ART at first ANC	11	23.4	57	32.4	68	30.5	20	37.7	142	53.2	162	50.6	< 0.0001	17	37.0	112	53.6	129	50.6	< 0.0001
Started ART first ANC (≤ 7 days)	29	61.7	90	51.1	119	53.4	30	56.6	102	38.2	132	41.3		26	56.5	81	38.8	107	42.0	
Started ART > 7 days firs ANC	7	14.9	29	16.5	36	16.1	3	5.7	23	8.6	26	8.1		3	6.5	16	7.7	19	7.4	
**First ART regimen**																				
TDF + 3TC + EFV	46	97.9	150	85.2	196	87.9	48	90.6	247	92.5	295	92.2	0.08	41	89.1	191	91.4	232	91.0	0.39
Other	1	2.1	26	14.8	27	12.1	5	9.4	20	7.5	25	7.8		5	10.9	18	8.6	23	9.0	
**Attended at least one HOPE visit**							46	86.8	209	78.3	255	79.7		46	100.0	209	100.0	255	100.0	
Median number of visits (IQR)							5 (4–8)		7 (5–9)		7 (5–9)			5 (4–8)		7 (5–9)		7 (5–9)		

^*^ Age group differences within period (pre and post) statistically significant *p* < 0.05

*TDF* tenofovir, *3TC* Lamivudine, *EFV* Efavirenz

### Incidence of LTFU

Incidence rates of LTFU over the first six months after enrollment in ANC services among women in the pre- and post-periods are shown in Table 2, overall and by age group. Among women < 20 years, LTFU in the pre-period was 2.36 per 100 person months (95%CI 1.06–5.25) (14.2% LTFU by six months) and 1.41 per 100 person months (95%CI 0.53–3.77) (8.5%) in the post-period. LTFU rates among women 20–24 years remained the same across the pre- and post-periods at 1.55 per 100 person months (95%CI pre: 0.93–2.57; post: 1.01–2.34) (9.3%). For post-period women who attended at least one HOPE session, the rate of LTFU in those < 20 years of age was 0.78 per 100 person years (95%CI 0.19–3.11) (4.7%) and for women 20–24 years, it was 1.13 per 100 person years (95%CI 0.65–1.94) (6.8%) (Table 2).

**Table 2:** Incidence rates, loss to follow-up among adolescent and young women living with HIV (AYWLH) < 25 years of age newly enrolled in antenatal care (ANC) services with antiretroviral therapy (ART) start date at seven HOPE project facilities in Kenya (*N* = 543)

	**Number loss to follow-up**	**Person months per group**	**Incidence rate per month**	**Incidence per 100 person months**	**95%CI**	**Proportion LTFU at six months**
**Pre-period (*N* = 223)**						
< 20 years	6	255	0.0236	2.36	1.06–5.25	14.2
20–24 years	15	969	0.0155	1.55	0.93–2.57	9.3
Total	21	1224	0.0172	1.72	1.12–2.63	10.3
**Post-period all women (*N* = 320)**						
< 20 years	4	283	0.0141	1.41	0.53–3.77	8.5
20–24 years	22	1430	0.0154	1.55	1.01–2.34	9.3
Total	26	1713	0.0152	1.52	1.03–2.23	9.1
**Post-period HOPE attendees (*N* = 255)**						
< 20 years	2	257	0.0078	0.78	0.19–3.11	4.7
20–24 years	13	1153	0.0113	1.13	0.65–1.94	6.8
Total	15	1410	0.0106	1.06	0.64–1.76	6.4

In unadjusted Poisson models comparing women in the pre- and post-period cohorts (including those who did not attend HOPE), among women < 20 years of age, the rate of LTFU in the first six months after enrollment in ANC was not significantly different (incidence rate ratio (IRR) 0.60, 95%CI 0.30–1.19). In adjusted models, adolescents in the post-period had lower rates of LTFU compared to the pre-period; however, this finding did not meet the threshold of statistical significance (adjusted IRR (aIRR) 0.62, 95%CI 0.38–1.01, *p* = 0.057) (Table 3). Among women 20–24 years, there was no statistically significant reduction in LTFU between the pre- and post-periods in either univariable or adjusted models (IRR 0.99, 95%CI 0.55–1.81; aIRR 1.18, 95%CI 0.72–1.94) (Table 3). Known HIV-positive status at first ANC visit was protective against LTFU in the first six months in both age groups, and among women 20–24 years, being on ART at the first ANC visit was also associated with lower LTFU (aIRR 0.66, 95%CI 0.44–0.98).

**Table 3:** Incidence rate ratios (IRR) for loss to follow-up in first 6 months after first ANC among adolescent and young women living with HIV (AYWLH) < 25 years among those with antiretroviral therapy (ART) start dates by age group, Kenya (*N* = 543)

	**AYWLH < 20 years of age**			**AYWLH 20–24 years of age**		
	**Adjusted incidence rate ratio**	**95%CI**	***p*-value**	**Adjusted incidence rate ratio**	**95%CI**	***p*-value**
**Pre-period vs. Post-period (all post-period AYWLH)**						
**Post-period (ref: pre-period)**	**0.62**	**0.38–1.01**	**0.057**	**1.18**	**0.72–1.94**	**0.50**
Gestational age weeks	0.96	0.88–1.05	0.42	0.99	0.97–1.02	0.65
Parity 1 + (ref: 0)	0.16	0.02–1.12	0.07	1.02	0.48–2.16	0.96
Known HIV + at first ANC (ref: newly diagnosed)	0.08	0.02–0.37	< 0.01	0.40	0.15–1.04	0.06
On ART at first ANC (ref: started ART first ANC)	2.83	0.62–12.98	0.18	0.66	0.44–0.98	0.04
**Pre-period vs. Post-period (women attending HOPE)**						
**Post-period (ref: pre-period)**	**0.41**	**0.08–2.05**	**0.28**	**0.85**	**0.46–1.57**	**0.60**
Gestational age weeks	0.98	0.90–1.08	0.71	0.99	0.96–1.01	0.30
Parity 1 + (ref: 0)	0.23	0.02–2.09	0.18	0.98	0.41–2.35	0.97
Known HIV + at first ANC (ref: newly diagnosed)	–	–	–	0.52	0.16–1.71	0.29
On ART at first ANC (ref: started ART first ANC)	1.89	0.19–18.98	0.59	0.50	0.31–0.80	< 0.01

Adjusted models for women < 20 years did not include a corrected for clustering within facility as sparse did not allow for model convergence; 95%CI: 95% confidence interval (adjusted for all variables included listed and intrasite clustering by facility); – indicates estimates that could not be generated due to sparse data

### Infant testing outcomes

Overall, there were 505 AYWLH with infants included in the analysis of HEI testing, 223 (100.0%) from the pre-period and 282 (88.1%) from the post-period (Table 4). In the pre-period cohort, 153 (68.6%) infants received 6 week EID testing compared to 218 (77.3%) in the post-period cohort indicating a 10% overall increase in infant testing (adjusted risk ratio (aRR) 1.10, 95%CI 1.01–1.21).

**Table 4:** Adolescent and young women living with HIV (AYWLH) < 25 years of age whose infants received 6–8 week PCR HIV testing and test results in the pre- and post-periods in Kenya (among those eligible^a^) (*N* = 514)

	HIV-exposed infants (HEI) tested 6–8 weeks											
	Pre-period		Post-period (All)		Adjusted risk ratio	95% CI	*p*-value	Post-period (HOPE)		Adjusted risk ratio	95%CI	*p*-value
	N	%	N	%				N	%			
< 20 years	47		50					43				
Infants tested	30	63.8	36	72.0	1.11	0.89–1.39	0.35	33	76.7	1.19	0.93–1.53	0.17
20–24 years	176		232					180				
Infants tested	123	69.9	182	78.4	1.10	1.02–1.19	0.01	152	84.4	1.19	1.13–1.26	< 0.0001
All	223		282					223				
Infants tested	153	68.6	218	77.3	1.10	1.01–1.21	0.04	185	83.0	1.20	1.10–1.31	< 0.0001
	HIV-exposed infants (HEI) PCR Test Results											
	Pre-period		Post-period (All)		Post-period (HOPE)							
	N	%	N	%	N	%						
< 20 years	30		36		33							
Positive	0	0.0	1	2.8	1	3.0						
Negative	28	93.3	34	94.4	32	97.0						
Unknown	2	6.7	1	2.8	0	0.0						
20–24 years	123		182		152							
Positive	2	1.6	3	1.7	3	2.0						
Negative	118	95.9	174	95.6	145	95.4						
Unknown	3	2.4	5	2.8	4	2.6						
All	153		218		185							
Positive	2	1.3	4	1.8	4	2.2						
Negative	146	95.4	208	95.4	177	95.7						
Unknown	5	3.3	6	2.8	4	2.2						

^a^Infants estimated to be at least 6 weeks of age at the time of data collection

^**^p-values for the comparison of testing of HEI in the pre vs. post period are from modified Poisson (relative risk) regression models adjusted for the following variables measured at the first antenatal care visit: age, gestational age in weeks, parity, known HIV-positive status and on ART

## Discussion

Following the introduction of a group antenatal care model for pregnant adolescents and young women living with HIV in Kenya, we observed a reduction in the incidence of LTFU at six months from 2.36 per 100 person months to 1.41 per 100 person months among women < 20 years of age. Although we observed increased retention among pregnant adolescents during the HOPE intervention, our sample of adolescents was small, and our findings did not meet the threshold for statistical significance (*p* = 0.057). HEI testing at 6–8 weeks was significantly higher following our intervention. We found that for women 20–24 years of age there was a 10% increase in EID overall and a 20% increase among women who attended HOPE. Given the paucity of data on interventions for pregnant and postnatal AYWLH, we believe the HOPE group model warrants further study as an approach to care for this highly vulnerable group.

Effective strategies for improving retention of adolescent pregnant and postnatal AYWLH are urgently needed [6–8, 10, 19] and we believe that our findings provide evidence that models providing adolescent-friendly services focused on the psychosocial needs of this vulnerable population warrant further examination. Using data from previous studies on barriers to care for adolescents living with HIV [14], HOPE was designed to foster social support through the group visit model, to engage women to participate in their own healthcare through self-care and to make services more adolescent-friendly for young mothers. We found an almost 40% reduction in LTFU. And, while our study was underpowered to detect a significant effect in this age group, our findings suggests that this model may hold promise for improving retention.

The HOPE model did not appear to improve retention among young women living with HIV 20–24 years suggesting that they have different needs that were not addressed by our intervention. In both the pre- and post-period cohorts, women 20–24 years were significantly more likely to have children, be married, and to know their status. HOPE services did, however, significantly improve uptake of HEI testing among women 20–24 years which is very encouraging as early diagnosis and immediate ART initiation are critical for improving survival among infants with HIV infection [20]. Among the adolescents, two-thirds (64%) in the pre-period and 77% in the post-period were having their first child, and most adolescents in both periods were newly diagnosed at first ANC. These very young mothers may have benefited specifically from the social support offered through HOPE services which may explain the discrepant findings across age groups and suggests that our intervention may be better suited to meet the needs of pregnant adolescents living with HIV. Other interventions will be needed to retain women 20–24 years. An additional finding of our study was that known HIV-positive status at the first ANC visit was protective against LTFU in both age groups. These data suggest that enhanced support services are needed for adolescent and young pregnant women at the time of HIV diagnosis to help them accept their status and receive the care they need.

Our evaluation has several important strengths, including our focus on identifying a model of care to meet the needs of pregnant AYWLH, an overlooked but vulnerable group, and our findings from routine care settings. The project was conducted in the same types of health facilities where most women accessing PMTCT in sub-Saharan Africa receive care, and services were delivered by facility staff nurses rather than trained research staff. Despite the common space and resource constraints that many health facilities face in similar settings, the project sites were able to conduct the services and show some impact from the intervention. We believe this is a strength as it indicates that this intervention could be undertaken in other resource-limited settings. Finally, few studies have reported retention estimates for adolescents, as such our study provides important new information. A 2018 systematic review of PMTCT retention in Option B + settings reported six-month retention from nine studies ranging from 47 to 88% (pooled estimate of 72.9%, 95%CI 66.4–78.9) but did not provide age-specific estimates [21]. The lack of retention data for AYWLH in PMTCT services is concerning given their higher risk for poor outcomes [9].

There are also limitations, including that almost 37% of women in the pre-period and 29% in the post-period were excluded from our analysis due to lack of ART data. The purposive approach to site selection which included mostly large health facilities may have contributed to this issue as women may attend only their first ANC visits at these sites as they offer more advanced care, including onsite laboratory services, than many lower level clinics which are attended for follow-up visits. The lack of documentation of these patterns of service utilization is a barrier to understanding outcomes among all women attending ANC and PMTCT services which has been previously noted [22, 23]. As a result of the missing outcomes of these women and because our study relied on routinely collected information from ART registers which may have incomplete data, it is possible that we have overestimated true LTFU among all AYWLH who attended a first ANC visit. A recent meta-analysis of tracing studies of PLHIV identified that women are more likely to have undocumented or ‘silent’ transfer; however this was not assessed by pregnancy status [24]. Our analysis was also limited to six-month retention after ANC entry whereas national guidelines call for women to remain in PMTCT services for up to 24 months. Longer term outcome data are needed to understand whether this intervention could retain pregnant and postnatal AYWLH for this period. Our study also used a pre-period comparison group and we cannot assess the extent to which improvements in PMTCT services between the pre- and intervention periods may have contributed to the reductions observed in LTFU in the latter period. In addition, higher proportions of women had known HIV-positive status and were already on ART at the first ANC visit in the intervention period, and these factors were found to be protective against LTFU. While we accounted for these differences in our adjusted models to isolate the effect of the intervention, these findings suggest positive trends in knowledge of HIV-positive status and ART initiation among adolescent and young women in Kenya from 2016 to 2019. Finally, our small sample size of only 100 adolescents limited our ability to measure statistically significant findings, however our effect estimate was large (aIRR 0.62) and our confidence interval (0.38–1.01) suggests that the intervention very likely had a protective effect. We hope that future studies using more robust designs, including cluster randomized trials, will be conducted to evaluate the impact of group antenatal care and enhanced adolescent-friendly PMTCT services.

## Conclusions

Interventions are urgently needed to improve outcomes among pregnant and postnatal AYWLH. We provide qualified evidence for an intervention that increased uptake of early infant diagnosis and may improve early retention in newly enrolled AYWLH in PMTCT services. Further evaluation are needed of DSD models similar to Project HOPE that support the health and psychosocial needs of this highly vulnerable group are needed.

## Data Availability

The routinely used medical record data utilized for the analyses presented in this study are owned by the Government of Kenya which has not given permission for them to be made publicly available. The datasets used and/or analysed during the current study are available from the corresponding author on reasonable request. Please send requests to ICAP-Columbia University: ct116@columbia.edu.

## Abbreviations

ANC: Antenatal care
AYWLH: Adolescents and young women living with HIV
ART: Antiretroviral therapy
CI: Confidence interval
DSD: Differentiated service delivery
HEI: HIV-exposed infant
IRR: Incidence rate ratio
aIRR: Adjusted incidence rate ratio
LTFU: Loss to follow-up
MCH: Maternal child health
NASCOP: National AIDS and STI Control Program
PMTCT: Prevention of mother-to-child transmission
PRC: Polymerase chain reaction
RR: Risk ratio
aRR: Adjusted risk ratio
SOC: Standard of care
WLHIV: Women living with HIV
IQR: Interquartile range
CDC: US Centers for Disease Control and Prevention
